# Expansion of Monocytic Myeloid-Derived Suppressor Cells in Patients Under Hemodialysis Might Lead to Cardiovascular and Cerebrovascular Events

**DOI:** 10.3389/fimmu.2020.577253

**Published:** 2021-01-29

**Authors:** Yan-Fang Xing, Jia-Rong Cai, Jun-Jian Qin, Wen-Ying Zhou, Can-Ming Li, Xing Li

**Affiliations:** ^1^ Department of Nephrology, The Third Affiliated Hospital of Guangzhou Medical University, Guangzhou, China; ^2^ Department of Urology, The Third Affiliated Hospital of Sun Yat-sen University, Guangzhou, China; ^3^ Department of Central Laboratory, The Third Affiliated Hospital of Sun Yat-sen University, Guangzhou, China; ^4^ Department of Nephrology, The Third Affiliated Hospital of Sun Yat-sen University, Guangzhou, China; ^5^ Department of Medical Oncology and Guangdong Key Laboratory of Liver Disease Research, The Third Affiliated Hospital of Sun Yat-sen University, Guangzhou, China

**Keywords:** monocytic myeloid-derived suppressor cell, hemodialysis, end stage renal disease, cardiovascular diseases, arginase

## Abstract

**Background:**

The specific mechanism of cardiovascular and cerebrovascular vasculopathy in the context of end-stage renal disease has not been elucidated. In the present study, we investigated the clinical impact of myeloid-derived suppressor cells (MDSCs) on hemodialysis patients and their mechanism of action.

**Methods:**

MDSCs were tested among 104 patients undergoing hemodialysis and their association with overall survival (OS) and cardiovascular and cerebrovascular events was determined.

**Results:**

Hemodialysis patients presented a significantly higher level of monocytic MDSCs (M-MDSCs) compared to healthy controls. M-MDSC were tested 3 months after first testing among 103 hemodialysis patients, with one patient not retested due to early death. The repeated results of M-MDSC levels were consistent with the initial results. Patients with persistent high level of M-MDSCs presented decreased OS, as well as increased stroke and acute heart failure events. As illustrated by multivariate Cox regression, M-MDSC was an independent predictor for OS and stroke events of hemodialysis patients. T cell proliferations were significantly abrogated by hemodialysis-related M-MDSCs in a dose-dependent manner. Besides, M-MDSCs presented higher levels of CXCR4 and VLA-4 compared to monocytes, which indicated their enhanced capability to be recruited to atherosclerotic lesions. The expression of arginase I and activity of arginase was also significantly raised in hemodialysis-related M-MDSCs. Human coronary arterial endothelial cells (HCAECs) presented increased capability to migration by coculture with M-MDSCs, compared with monocyte group. Arginase inhibitor and L-arginine abrogated the immune suppressive function and induction of HCAECs migration of hemodialysis related M-MDSC. Plasma IFN-*γ*, TNF-α and IL-6 were elevated in hemodialysis patients compared with healthy control. M-MDSC level was positively related to IL-6 level among hemodialysis patients. The plasma of hemodialysis patients induced M-MDSCs significantly compared with plasma from health donors. Besides, IL-6 neutralizing antibody significantly abrogated the induction. Neutralizing antibody of IFN-*γ* and TNF-α partially decreased the generation of arginase of the induced M-MDSC.

**Conclusions:**

M-MDSCs were elevated in ESRD patients under hemodialysis, and they exhibited a strong association with the risk of cardiovascular and cerebrovascular diseases. Hemodialysis related M-MDSC presented enhanced recruitment to atherosclerotic lesions, promoted the migration of endothelial cells through exhaustion of local L-arginine.

## Introduction

Cardiovascular and cerebrovascular diseases are the top causes of death for end-stage renal disease (ESRD) patients and contribute to approximately half of the mortality rate (1, 2). The latent mechanism of cardiovascular and cerebrovascular diseases among ESRD patients is different from that among the general population (3). ESRD patients exhibit reverse associations with traditional risk factors of cardiovascular diseases compared to the general population (3). In contrast to the general population, obesity, hypercholesterolemia, and hypertension paradoxically appear to be protective features (3). Therefore, the specific mechanism of cardiovascular vasculopathy in the context of ESRD needs to be investigated.

Macrophages in atherosclerotic lesions are partially derived from monocytes recruited by the pathologic endothelial cell layer of blood vessels. They participate in a maladaptive, non-resolving inflammatory response that expands the subendothelial layer, which subsequently leads to necrotic cores and development of acute thrombotic vascular disease (4, 5). In our previous study, we found that HLA-DR^−/low^CD11b^+^CD14^+^CD15^−^ cells were increased in ESRD patients after hemodialysis (6), and they could be monocytes or monocytic myeloid-derived suppressor cells (M-MDSCs) (7, 8). Monocytes and M-MDSCs were believed to transform into macrophages in multiple backgrounds, including tumor microenvironments (8, 9). Thus, we speculated that hemodialysis-related HLA-DR^−/low^CD11b^+^CD14^+^CD15^−^ cells might contribute to the vasculopathy for ESRD patients.

In the present study, we found that hemodialysis-related HLA-DR^−/low^CD11b^+^CD14^+^CD15^−^ cells were M-MDSCs with immune suppression capability, and were correlated with cardiovascular and cerebrovascular diseases and hazarded the overall survival (OS) of hemodialysis patients. Hemodialysis related M-MDSC presented enhanced recruitment to atherosclerotic lesions, promoted the migration of endothelial cells through exhaustion of local L-arginine, which might be associated with the mechanism of ESRD related atherosclerosis.

## Materials

### Selection of Patients and Healthy Donors

All ESRD patients undergoing hemodialysis and healthy controls were screened for serum HIV antibody, hepatitis B surface antigen (HBsAg), hepatitis C virus (HCV) antibody, hepatitis D virus (HDV) antigen, and HDV antibody. Patients and healthy controls who were positive for HIV tests, had chronic hepatitis virus infection, were pregnant, received systematic corticosteroids or immunosuppressive agents, presented a fever or acute infections (*e.g.*, pneumonia or urinary tract infection) within one week before recruitment, as well as those short of fundamental baseline data or reluctant to participate were excluded from this study (**Figure S1**). The study was approved by the Clinical Ethics Review Board of the Third Affiliated Hospital of Guangzhou Medical University and the Third Affiliated Hospital of Sun Yat-sen University. A written informed consent was obtained from all patients at the time of recruitment.

Baseline characteristics related to the prognosis or immunity of hemodialysis patients were collected, such as age, gender, white blood cell count, red blood cell count, neutrophil count, monocyte count, and so on, before initiation of hemodialysis and MDSC testing. For patients reluctant to pay for the above-mentioned routine tests, the tests were paid for by the funds for the present study.

### Flow Cytometric Analysis

The cell phenotypes were analyzed by flow cytometry on the FACSAria II flow cytometer (BD Biosciences), and data were analyzed with the FlowJo V10.0.7 (FlowJo, Ashland, OR). For flow cytometric sorting, the BD FACSAria cell sorter (BD Biosciences) was used. The strategy for M-MDSC sorting was selection of CD11b^+^CD14^+^HLA-DR^−/low^CD15^−^ from live peripheral blood mononuclear cells (PBMCs), with selection of CD3^+^ T cells from live PBMCs. For intracellular cytokine staining, PBMCs were stimulated in complete RPMI 1640 (Life Technologies) with 50 ng/ml PMA (Sigma-Aldrich), 1 mg/ml ionomycin (Sigma-Aldrich), and 1 mg/ml brefeldin A (Invitrogen) for 4 h. Cells were then stained with the Abs against surface markers, fixed, and permeabilized using an Intracellular Fixation and Permeabilization Buffer Set (eBioscience). After that, cells were stained with the antibodies against cytokines. The experiments were performed in a biosafety laboratory. Agents used were listed in **Table S1**.

### T Cell Proliferation and Activation Assay

T cell proliferation was determined by CFSE (5,6-carboxyfluoresceindiacetate, succinimidylester) dilution. T cells were sorted from hemodialysis patients and/or healthy donor, and used for further analysis to avoid the heterogeneity in proliferation capacity of T cells from different donors (10, 11). Purified T cells were labeled with CFSE (3 mM; Invitrogen, Carlsbad, CA), stimulated with 0.5 mg/ml of pre-coated anti-CD3 and 0.5 mg/ml anti-CD28 (eBioscience), then cultured either alone or co-cultured with M-MDSCs at the indicated ratios for three days. The cells were then stained for surface marker expression with CD4-PE or CD8-APC antibodies, and T cell proliferation was analyzed on a flow cytometer. All cultures were carried out in the presence of 20 IU/ml recombinant human IL-2 (PeproTech, Rocky Hill, NJ) in RPMI 1640 (Life Technologies, Carlsbad, CA) for three days at 37°C. Where indicated, 1 mM L-arginine, 0.5 mM nor-NOHA (an arginase I specific inhibitor; Cayman Chemicals, Ann Arbor, MI), or 100 μM L-NMMA (an iNOS inhibitor; Cayman Chemicals, Ann Arbor, MI), was added to the culture on day zero.

### ELISA

Culture supernatants of the T cell and MDSC co-culture system, plasma of patients and donors were collected for ELISA testing. Interferon-*γ* (IFN-*γ*), IL-6, and tumor necrosis factor-α (TNF-α) quantification in the supernatants was determined by an enzyme-linked immunosorbent assay (ELISA) following the manufacturer’s instructions (**Table S2**).

### Arginase Activity Assay

The activity of arginase was measured in cell lysates. Briefly, cells were lysed with 0.1% Triton X-100 for 30 min, then 25 mM Tris-HCl and 10 mM MnCl_2_ were added. The enzyme was activated by heating at 56°C for 10 min. Arginine hydrolysis was performed by incubating the lysate with 0.5 M L-arginine for 120 min at 37°C. After the addition of a-isonitrosopropiophenone (dissolved in 100% ethanol), the urea concentration was measured at 540 nm, followed by heating at 95°C for 30 min.

### Nitric Oxide Assay

The nitric oxide (NO) content in plasma was measured using the QuantiChrom Nitric Oxide Assay Kit, following the manufacturer’s instructions (BioAssay Systems, Hayward, CA). Culture media samples (150 μl) were first mixed with ZnSO4 (8 μl) and vortexed; then NaOH (8 μl) was added, followed by centrifugation for 10 min at 14,000 rpm. 100 μl of the deproteinized supernatants was transferred to a clean tube, mixed with a combination of 100 μl of Reagent A, 4 μl of Reagent B, and 100 μl of Reagent C, and incubated for 10 min at 60°C. The absorbance at 570 nm was measured using a microplate reader (Bio-Rad, Hercules, CA). Nitrite concentrations were determined by comparing the absorbance values for the test samples to a standard curve generated by serial dilution of 0.25 mM sodium nitrite.

### Quantitative Reverse Transcription Polymerase Chain Reaction

RNA was extracted with the Multisource Total RNA Miniprep Kit (Axygen, Union City, CA), and quantitative reverse transcription polymerase chain reaction was performed utilizing commercially available primers (**Table S3**) and SYBR Premix ExTaq II (Code, DRR081; Takara Biotechnology Co., Dalian, China). Fluorescence for each cycle was quantitatively analyzed using the ABI Prism 7000 sequence detection system (Life Technologies). The results were reported as relative expression, normalized with the GAPDH housekeeping gene as an endogenous control. Monocytes from one health donor were used as control and compared by the monocytes from other health donors and M-MDSC from hemodialysis patients. The relative mRNA expression was displayed in arbitrary units.

### Migration Assays

Human coronary arterial endothelial cells (HCAECs) (Lonza, Basel, Switzerland) were cultured in EGM-2 media (Lonza). Cell migration was assessed using a 24-well QCM Chemotaxis cell migration assay (Millipore) with 8 mm pore size as per the manufacturer’s instructions. Cells were resuspended in EBM-2 serum-free medium, counted and plated onto the up chamber at 37,500 cells per chamber in triplicate. Cells were allowed to migrate overnight towards EBM-2 media + 10% FBS at 37°C. Monocytes from health donors, M-MDSCs from hemodialysis patients were added at a 1:10 ratio (M-MDSCs: HCAECs), with 1 mM L-arginine or 0.5 mM nor-NOHA administrated as indicated. The cells in the lower chambers were then fixed with 4% paraformaldehyde for 10 min, washed once with PBS solution, stained with crystal violet for 10 min, washed again with PBS solution, and finally counted and photographed with a Leica DC300F digital microscope (Leica Camera AG, Solms, Germany).

### *In Vitro* Monocytic Myeloid-Derived Suppressor Cells Generation From Peripheral Blood Mononuclear Cell

PBMCs from health donor cells were cultured in RPMI 1640 medium supplemented with 10% FBS, 20 ng/ml GM-CSF, and 50 µM 2-ME. The cultures were maintained at 37°C in 5% CO_2_-humidified atmosphere in 24-well plates. Medium was refreshed on day 3. Cells were analyzed by flow cytometry on day 5. 20 ng/ml of IL-6 neutralizing mAb (IL-6 mAb, R&D Systems), 10 μg/ml of infliximab (MSD, Whitehouse Station, NJ) or 1 µg/ml of both anti-IFN-*γ* (R&D Systems, MAB285, clone # 25718) and anti-IFN-*γ*R1 (R&D Systems, MAB6731, clone # 92101) was added with PBS as vehicle control.

### Patient Follow-Up and Statistical Analysis

Patients returned for follow-up appointments at least every week until death. The follow-up duration was calculated from the first day of M-MDSC testing to the day of death, or to the last follow-up. OS was the end point, which was calculated from the first day of treatment to death (12).

Variables in different groups were compared using the χ² test (or the Fisher's exact test, if indicated) and t-test or the non-parametric Mann–Whitney U tests. OS was calculated using the Kaplan–Meier survivor function and with Kaplan–Meier failure function conducted to illustrate occurrence of stroke events, acute myocardial infarction (AMI) events, and heart failure events since the day of M-MDSC testing. Their differences were compared using the log-rank test. Multivariate analysis using a Cox proportional hazards model was used to test for independent significance by entry of insignificant explanatory variables. Covariates including host factors (*i.e.*, age and gender), characteristics of MDSC testing, and MDSC subsets were included in all tests. For *in vitro* experiments, statistical analyses were done using paired t-tests. Correlations between different parameters were analyzed using the Spearman’s rank test (13). The criterion for statistical significance was set at *α* = 0.05 and all *P*-values were based on two-sided tests. Statistical tests were performed using GraphPad Prism version 5.0a and SPSS Statistics 20.0. P values of 0.05 were considered significant.

## Results

### Monocytic Myeloid-Derived Suppressor Cells Were Elevated in Hemodialysis Patients and Decreased Overall Survival

During the period between October 2015 and December 2015, we investigated a series of 104 ESRD patients undergoing maintenance hemodialysis in the Third Affiliated Hospital of Guangzhou Medical University and the Third Affiliated Hospital of Sun Yat-sen University, Guangzhou, China. The ESRD was considered irreversible by two independent nephrologists rather than representing acute kidney injury. Age- and gender-matched healthy controls (n = 60) consisted of local volunteers. The complications of the 104 hemodialysis patients when testing M-MDSC were documented, which revealed that 36 patients presented diabetes, 31 presented stable coronary heart disease, 72 patients presented chronic heart failure, and 30 patients presented cerebrovascular disease. A total of 37 patients died before the ending of this study. The major death causes were heart diseases and stroke (**Table S4**). The complications of the health donor were all stable, with none of them presented poorly controlled diseases such as diabetes, hypertension, cardiovascular disease, and cerebrovascular disease (**Table S5**).

**Figure 1:**
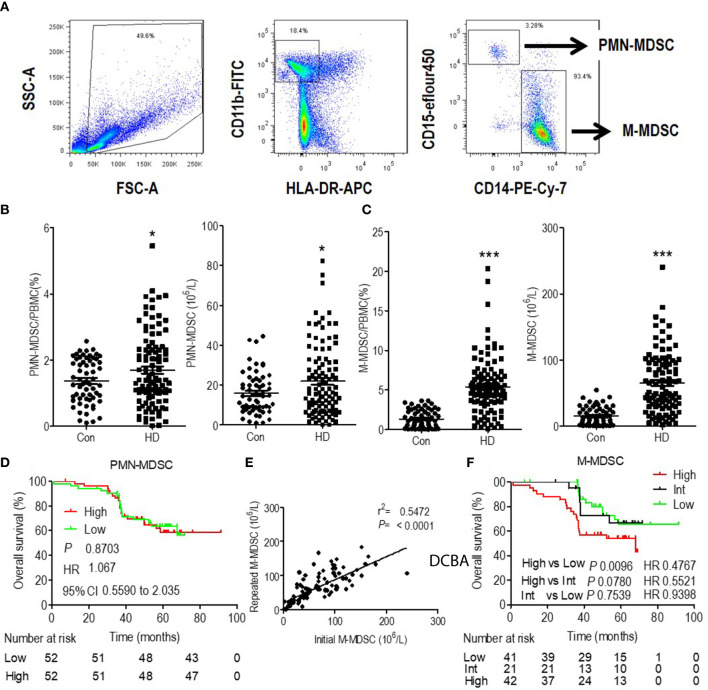
Expansion and prognostic value of monocytic myeloid-derived suppressor cells (M-MDSCs) in end-stage renal disease (ESRD) patients undergoing hemodialysis. **(A)** Gating strategy of monocytic MDSCs (M-MDSCs) by flow cytometry analysis. Polymorphonuclear-MDSC (PMN-MDSC) was defined as CD11b^+^CD15^+^CD14^−^HLA-DR^−/low^, with M-MDSC defined as CD11b^+^CD15^−^CD14^+^HLA-DR^−/low^. Frequency and absolute count of PMN-MDSCs **(B)** and M-MDSCs **(C)** in the peripheral blood of healthy controls and ESRD patients under hemodialysis (HD). *P < 0.05, ***P < 0.001. **(D)** Kaplan–Meier survival curves are shown for overall survival in low and high levels of PMN-MDSC hemodialysis patients. **(E)** Linear regression analysis of the association between initial M-MDSC results and repeated M-MDSC results (3 months later). **(F)** Kaplan–Meier survival curves are shown for overall survival in low, intermediate (Int) and high levels of M-MDSC hemodialysis patients. For Kaplan–Meier survival curves, hazards ratios (HRs) were calculated using the unadjusted Cox proportional hazards model. *P* values were calculated using the unadjusted log-rank test. 95% CI indicates 95% confidence interval.

Blood samples were collected within 1 h before daily hemodialysis during maintenance hemodialysis. PBMCs were isolated from whole blood by Ficoll centrifugation and analyzed within 6 h of blood sampling. Circulating frequencies of the subsets of MDSCs were quantified with the gating strategy indicated. M-MDSCs were HLA-DR^−/low^CD11b^+^CD14^+^CD15^−^ and polymorphonuclear-MDSCs (PMN-MDSCs) were HLA-DR^−/low^CD11b^+^CD14^−^CD15^+^ (**Figure 1A**). Patients with ESRD undergoing hemodialysis presented slightly increased PMN-MDSCs (**Figure 1B**) and a significantly higher level of M-MDSCs (**Figure 1C**) compared to that in healthy controls.

Multivariate Cox analysis revealed that M-MDSC and age were the independent prognostic factors for OS among hemodialysis patients (**Table S6**). Besides, PMN-MDSC levels were dichotomized to high and low level according to the median value. Kaplan–Meier analysis revealed that they were not associated with OS. (**Figure 1D**). Thus, we focused on M-MDSC in further analysis.

M-MDSC were tested 3 months after first testing among 103 hemodialysis patients, with one patient not retested due to early death. The repeated results of M-MDSC levels were consistent with the initial results (**Figure 1E**). M-MDSC levels were dichotomized to high and low levels according to the median value of each testing. Among the 103 patients, 21 changed their category of M-MDSC levels (Intermediate group). M-MDSC levels of 10 patients changed from high level in the initial test to low level in the second test, with the rest 11 patients changed reversely. 41 patients displayed persistent low M-MDSC levels, with the rest 42 patients presenting persistent high M-MDSC levels (**Figure S2**). More patients died at the end of this study among the patients with high M-MDSC levels (p = 0.035) (**Table S4**). Patients with persistent high M-MDSC levels presented higher lymphocyte and monocyte counts and lower serum creatine when testing M-MDSC. They displayed better eGFR before initiation of hemodialysis (**Table S4**). Survival analysis revealed that patients with persistent high level of M-MDSC presented the worst OS (**Figure 1F**).

### Monocytic Myeloid-Derived Suppressor Cells Contributed to Hemodialysis-Related Cardiovascular Diseases

**Figure 2:**
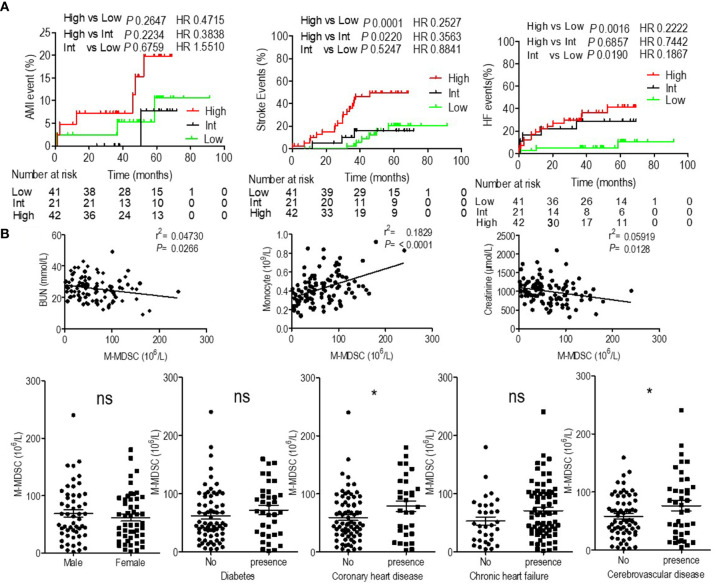
Prognostic value of monocytic myeloid-derived suppressor cells (M-MDSCs) for cardiovascular diseases in end-stage renal disease (ESRD) patients under hemodialysis and their correlation with clinical characteristics. **(A)** Kaplan–Meier survival curves are shown for acute myocardial infarction (AMI), heart failure (HF), and stroke-free survival in low, intermediate (Int) and high levels of M-MDSCs hemodialysis patients. Hazard ratios (HRs) were calculated using the unadjusted Cox proportional hazards model. *P* values were calculated using the unadjusted log-rank test. **(B)** Correlation with clinical characteristics.

Patients with high levels of M-MDSCs presented decreased OS, but the reasons had to be identified. In our previous study we found that M-MDSCs did not influence the risk of infectious disease (6), which was the second leading cause of mortality for ESRD patients. Then, we analyzed the association of M-MDSCs with cardiovascular and cerebrovascular diseases, which were the major causes of mortality for ESRD patients (1, 2). Stroke, AMI, and acute heart failure events were documented. Kaplan–Meier failure analysis displayed that patients with persistent high M-MDSC levels presented significantly higher risk of stroke and heart failure than patients with low and intermediate M-MDSC levels. But M-MDSC levels did not influence AMI events (**Figure 2A**). Correlation analysis revealed that M-MDSC levels were positively related to blood urine nitrogen (BUN), monocyte count, and creatinine (**Figure 2B** and **Figure S3**). Hemodialysis patients with coronary heart disease and cerebrovascular disease presented significantly higher M-MDSC levels than those without the complications. Diabetes and chronic heart failure did not influence the M-MDSC levels (**Figure 2B**). Multivariate Cox regression revealed that M-MDSC level was an independent prognostic factor for stroke event, but not for heart failure and AMI (**Tables S7–S9**). Concerning other prognostic factors, age was related to AMI events (**Table S7**). Lower serum creatinine, higher serum albumin, and urea reduction rates predicted heart failure events (**Table S8**). Age, white blood cell counts, neutrophils counts, monocyte counts predicted stroke events (**Table S9**). Above all, M-MDSCs were confirmed to be a prognostic factor for cardiovascular and cerebrovascular diseases among hemodialysis patients, especially for stroke events.

### Monocytic Myeloid-Derived Suppressor Cells in Hemodialysis Patients Presented Immune Suppressive Function

**Figure 3:**
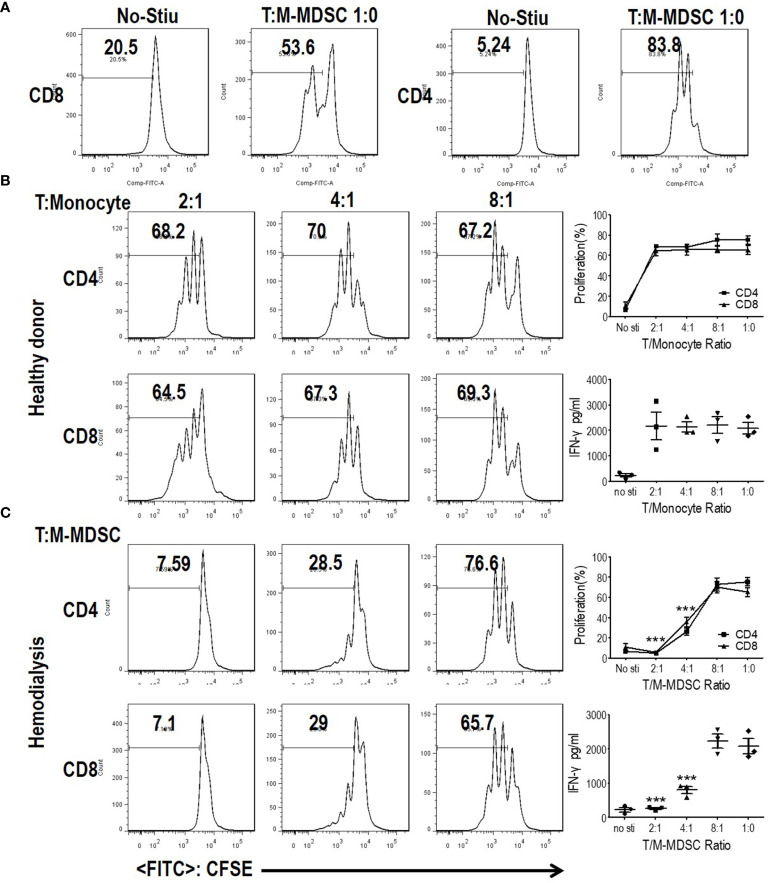
Monocytic myeloid-derived suppressor cells (M-MDSCs) from end-stage renal disease (ESRD) patients under hemodialysis suppressed T cell proliferation and activation. CD3^+^T cells from peripheral blood mononuclear cells (PBMCs) were stimulated with anti-CD3 and anti-CD28, co-cultured with M-MDSCs from the same donors at different ratios for 3 days, and evaluated for CD4^+^ and CD8^+^ T cell proliferation by carboxyfluorescein diacetate succinimidyl ester (CFSE) labeling and interferon-*γ* production in supernatants by enzyme-linked immunosorbent assay. **(A)** Representative flow cytometry data of positive control and negative control. **(B)** Results of healthy controls. **(C)** Results of patients with ESRD. Left panels: Representative flow cytometry data from one individual. Right panels: Cumulative data (n = 3) and concentration of interferon-*γ* in the media (n = 3). ***P < 0.001.

Since M-MDSCs share the same surface marker with monocytes, HLA-DR^−/low^CD11b^+^CD14^+^CD15^−^ cells in the present study might be M-MDSCs or monocytes (7). Thus, it was imperative to define whether these cells were M-MDSCs or monocytes. M-MDSCs are characterized by their suppressive capability on T cell response because they share the same definitive markers as their normal counterparts, monocytes (6–8, 14). To investigate whether M-MDSCs in ESRD patients undergoing hemodialysis suppressed T cell response, T cells and M-MDSCs were purified from PBMC using flow sorting. CFSE-labeled PBMC-derived CD3 T cells were stimulated with anti-CD3 and anti-CD28, with the indicated ratio of M-MDSCs in a co-culture system. CD4 and CD8 T cell proliferations were significantly abrogated by the addition of hemodialysis-related M-MDSCs in a dose-dependent manner. The IFN-*γ* levels in the media, tested using ELISA, illustrated that IFN-*γ* secretion was decreased after administration of hemodialysis-related M-MDSCs. Meanwhile, monocytes from healthy donors did not exhibit a suppressive function. (**Figures 3** and **S4A, B**) Then, cross-validation experiments with co-cultures were conducted to verify the results. Monocytes did not suppress the proliferation and activation of T cells from both health donors and hemodialysis patients. M-MDSC from hemodialysis suppressed T cells from both health donors and hemodialysis patients. (**Figures S5A–D**) T cells from hemodialysis patients did not proliferate after stimulation as well as those from the health donors (**Figures S5E, F**). M-MDSCs existed in ESRD patients with hemodialysis, but not in healthy donors. Above all, HLA-DR^−/low^CD11b^+^CD14^+^CD15^−^ cells in hemodialysis patients were M-MDSCs.

### Monocytic Myeloid-Derived Suppressor Cells Presented Higher Cell Surface Adhesion Molecules

**Figure 4:**
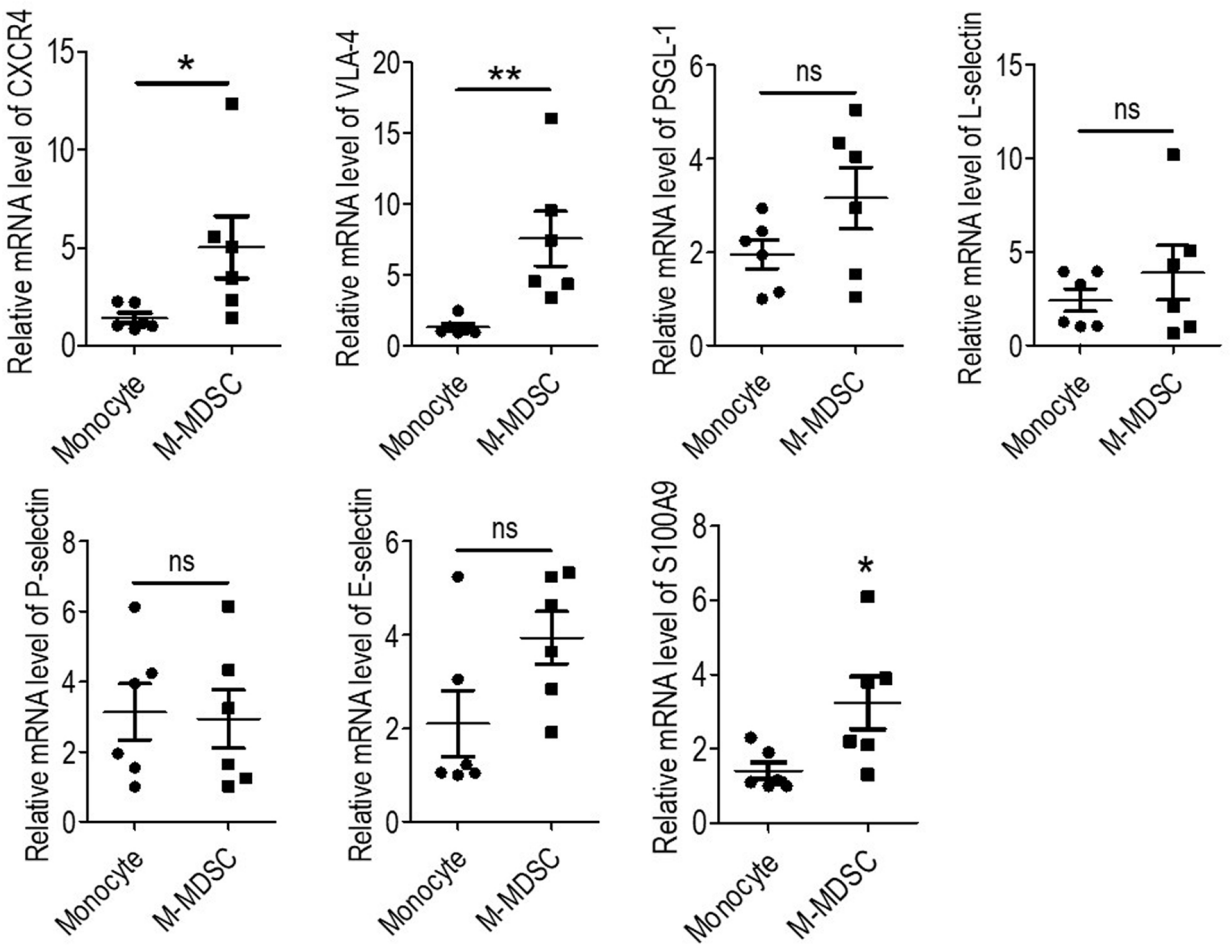
Expression of cell surface adhesion molecules and S100A9 of monocytic myeloid-derived suppressor cells (M-MDSCs). Expression of CXCR4, VLA-4, PSGL-1, L-selectin, P-selectin, E-selectin, and S100A9 were evaluated by qRT-PCR in M-MDSCs and monocytes. (n = 6). *P < 0.05; **P < 0.01.

Recruitment of monocytes to the vessel wall was reported to be an early step in the formation of atherosclerotic lesions (4, 15). Recruited monocytes induced local inflammation and were a source of foam cells (5). Thus, to investigate the recruitment behavior of M-MDSCs, the expression of adhesion molecules was investigated using qRT-PCR. As a result, M-MDSCs presented higher levels of CXCR4 and VLA-4 compared to those of monocytes, with similar levels of PSGL1, L-selectin, P-selectin, and E-selectin. Thus, compared to monocytes, hemodialysis-related M-MDSCs were more likely to be recruited to atherosclerotic lesions (**Figure 4**).

Elevation of S100A9 was reported to be an important up-stream molecular event for the development of M-MDSCs (7, 16, 17). S100A9 was tested in hemodialysis-related M-MDSCs and monocytes, thus revealing that S100A9 had significantly higher expression in M-MDSCs (**Figure 4**). This result indicated that S100A9 might induce the expression of CXCR4 and VLA-4.

### Monocytic Myeloid-Derived Suppressor Cells Induced Migration of Vascular Endothelial Cell by Arginine Deprivation

**Figure 5:**
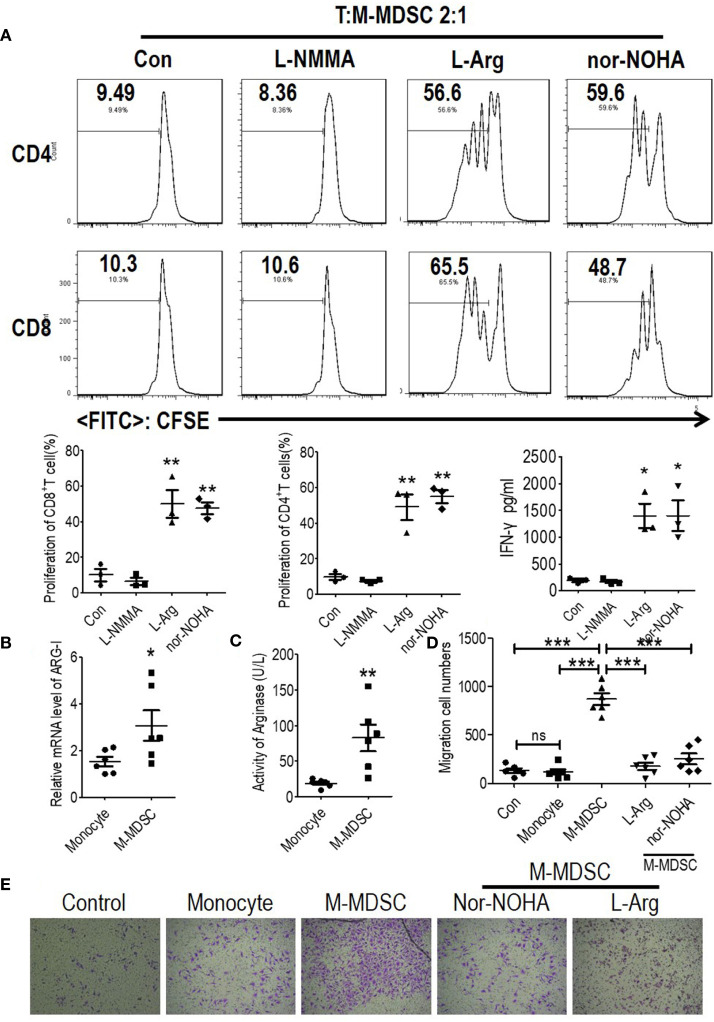
Monocytic myeloid-derived suppressor cells (M-MDSCs) suppressed functional T cells and promoted human coronary arterial endothelial cells (HCAECs) migration in an arginase 1-dependent manner. **(A)** Effect of arginase inhibitor NOHA, L-arginine supplementation, or inducible nitric oxide synthase (iNOS) inhibitor L-NMMA on M-MDSC function. T cells from ESRD patients were stimulated with anti-CD3/anti-CD28, cocultured with M-MDSCs from whole blood at a 2:1 ratio with treatments as indicated, evaluated for T cell proliferation by CFSE labeling, and IFN-*γ* production in supernatants by ELISA. Representative flow cytometry data, cumulative data (n = 3), and concentration of IFN-*γ* in the media (n = 3). Expression of Arg1 **(B)** and arginase activity **(C)** in M-MDSCs and monocytes. (n = 6). **(D, E)** The migration of HCAECs was tested using transwell experiment with monocytes from healthy donors, M-MDSCs from hemodialysis patients added at a 1:10 ratio (M-MDSCs: HCAECs). 1 mM L-arginine or 0.5 mM nor-NOHA was added as indicated in M-MDSCs’ treatment group. Cumulative data **(D)** and typical results were shown **(E)**. *P < 0.05; **P < 0.01; ***P < 0.001.

Recruited monocytes played vital roles in both early atherogenesis and advanced plaque progression by multiple mechanisms including transformation into macrophages (4, 5, 18). Thus, to investigate the mechanism of the M-MDSC-induced acceleration of cardiovascular disease, we tested the bioactive substances which were reported to be generated by M-MDSCs from a tumor background. Based on the finding that suppression of T cell proliferation was a feature of M-MDSCs differing from monocytes, we further explored the underlying mechanisms controlling hemodialysis-related M-MDSC-mediated T cell suppression. Previous reports confirmed that arginase I or NO were immune mediators for M-MDSC-mediated immune suppression (7, 8, 14, 19). Thus, in M-MDSCs and T cell proliferation (ratio 1:2) co-culture system, an arginase inhibitor (N *ω*-hydroxy-nor-l-arginine, nor-NOHA), L-arginine supplementation, or an inducible nitric oxide synthase (iNOS) inhibitor (N^G^-Monomethyl-L-arginine, L-NMMA) was utilized to reverse the suppressive effects of M-MDSCs on T cell proliferation in a co-culture system (20). Nor-NOHA, L-arginine and L-NMMA did not influence the proliferation of T cells (**Figure S6**). As a result, the suppression on T cell proliferation and IFN-*γ* production was reversed by arginase inhibitor, nor-HOHA, and L-arginine (**Figures 5A** and **S4C**). Meanwhile, the expression of arginase I and activity of arginase were also significantly raised in hemodialysis-related M-MDSCs compared to monocytes from healthy donors (**Figures 5B, C** **)**.

Decreased L-arginine was reported as critically important for endothelial nitric oxide synthase (eNOS) uncoupling, which led to decreased NO production, increased ROS generation, and finally the development of atherosclerosis (21, 22). Migration of vascular endothelial cells is a critical process for the development of atherosclerosis (23). In order to prove the M-MDSCs were involved in the development of atherosclerosis, M-MDSCs from hemodialysis patients were co-cultured with HCAECs. HCAECs presented increased capability to migration in M-MDSC group compared with monocyte group. Arginase inhibitor, nor-HOHA, and L-arginine reversed this phenomenon (**Figures 5D, E** **)**. Thus, M-MDSCs might promote the migration of endothelial cells into vascular intima through deprivation of L-arginine.

### Monocytic Myeloid-Derived Suppressor Cells Might be Induced by Elevation of Interferon-*γ*, Tumor Necrosis Factor-α, and IL-6 in Hemodialysis Patients

**Figure 6:**
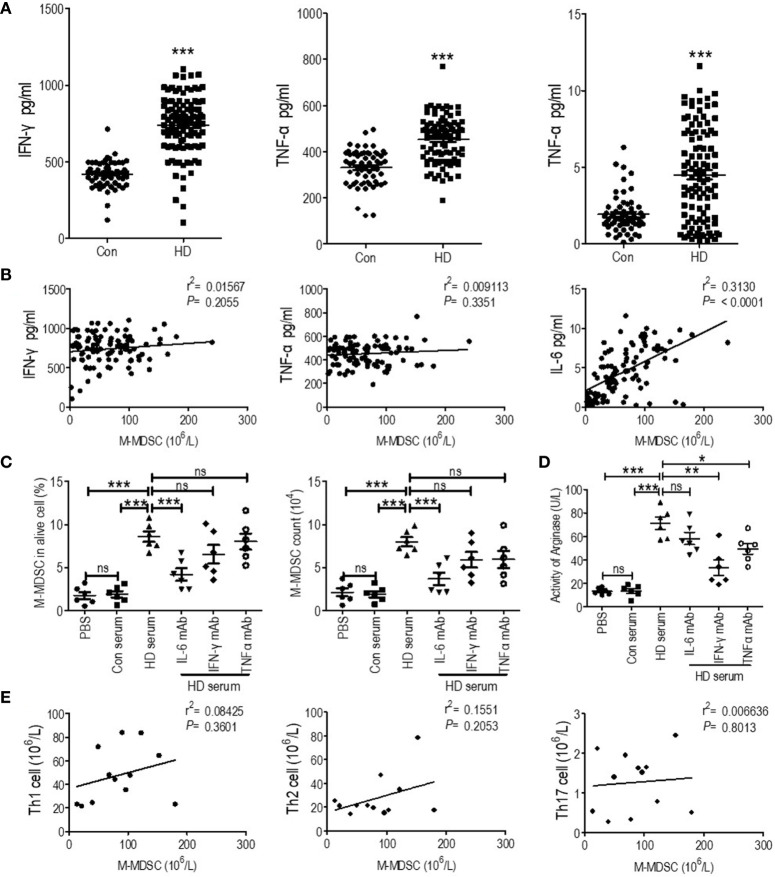
Correlation of plasma cytokines and T helper (Th) cells with M-MDSCs among end-stage renal disease (ESRD) patients under hemodialysis. **(A)** IFN-*γ*, TNF-α and IL-6 levels of the plasma collected when testing M-MDSC of health donor (Con) and hemodialysis patients (HD). **(B)** Linear regression analysis of the association between M-MDSC level and IFN-*γ*, TNF-α, and IL-6 levels. **(C, D)** Plasma of hemodialysis patients was utilized to induce M-MDSC from PBMC of healthy donor with or without neutralizing antibody of IL-6, IFN-*γ*, and TNF-α. The percentage in alive cells and absolute cell counts of M-MDSC **(C)** and the activity of arginase of induced M-MDSCs **(D)** in each group were shown (n = 6). **(E)** Linear regression analysis of the association between M-MDSC results and Th1, Th1, and Th17 levels (n = 12). *P < 0.05; **P < 0.01; ***P < 0.001.

According to previous reports, IFN-*γ* and TNF-α activated the immune suppressive function of M-MDSCs, and IL-6 induced M-MDSCs’ expansion (24). Thus, we test IFN-*γ*, TNF-α, and IL-6 in the plasma of hemodialysis patients collected when testing M-MDSCs. We found that plasma IFN-*γ*, TNF-α and IL-6 was elevated in hemodialysis patients compared with healthy controls (**Figure 6A**). Notably, linear regression found positive association between M-MDSC levels and IL-6 levels among hemodialysis patients (**Figure 6B**). IL-6 predicted OS, and IFN-*γ* predicted AMI events and stroke events. Then, plasma of hemodialysis patients and that from health donors was utilized to induce M-MDSCs from PBMCs of healthy donors. As a result, the plasma of hemodialysis patients induced M-MDSCs significantly compared with the plasma from healthy donors. Besides IL-6 neutralizing antibody partially abrogated the induction (**Figure 6C**). Neutralizing antibodies of IFN-*γ* and TNF-α partially decreased the generation of arginase of the induced M-MDSCs (**Figure 6D**). Thus, our results indicated that the elevation of plasma IL-6 in the hemodialysis patients might be one of the causes of M-MDSCs’ elevation, with the increase of IFN-*γ* and TNF-α associated with activation of M-MDSCs.

Besides, Th1, Th2, and Th17 cells were tested in 12 hemodialysis patients and 12 healthy control (**Figure S6A**). They were all elevated in hemodialysis patients (**Figure S7**). However, their levels were not associated with M-MDSC levels (**Figure 6E**).

## Discussion

Cardiovascular and cerebrovascular diseases are the leading cause of mortality for ESRD patients (1, 2). However, the mechanism of ESRD-related cardiovascular diseases for these patients was considered quite different from that of the general population. Interestingly, ESRD patients exhibit reverse associations with traditional risk factors for cardiovascular diseases. Hemodialysis was considered a critical cause of cardiovascular and cerebrovascular diseases due to severe hemodynamic changes (2). Obesity, hypercholesterolemia, and hypertension paradoxically appear to be protective features, opposite to the general population (3). Thus, the novel mechanism of ESRD-related cardiovascular diseases was investigated. In our present study, we found that M-MDSCs were elevated in hemodialysis patients. And persistent high M-MDSC levels predicted OS and cardiovascular and cerebrovascular disease events. Hemodialysis related M-MDSC presented enhanced recruitment to atherosclerotic lesions and exhaustion of local L-arginine, which might be involved in the mechanism.

Monocytes and M-MDSC share the same surface marker and morphology (7, 8, 25). Monocytes play a critical role in the development of cardiovascular diseases. They could migrate to atherosclerotic lesions, transform to foam cells, and induce local metabolic changes (4, 5). However, the predictive value of circulating monocyte count for cardiovascular diseases remains under debate (26–29). Some reports suggest that circulating monocyte count predicts cardiovascular diseases in ESRD patients (26–29). In our study, monocyte count was a potential predictor of stroke events among ESRD patients. However, blood cell count could not distinguish M-MDSCs from monocytes due to their similar morphology. Thus, it was necessary to identify the nature of these cells. This present study revealed they were M-MDSCs and presented independent prognostic value for OS and stroke events among hemodialysis patients.

Hemodialysis-related M-MDSCs were confirmed to suppress T cell proliferation. However, we could not conclude that M-MDSCs promoted cardiovascular and cerebrovascular diseases by induction of immune suppression. Immune suppression capacity illustrated their nature as M-MDSCs. However, current data did not support the immune suppressive function of M-MDSC induced cardiovascular and cerebrovascular events among hemodialysis patients. Previous studies on T cells of ESRD patients revealed presence of preactivated T cells in hemodialyzed patients (30). Consequently, T cell proliferation and activation in response to mitogen are impaired (31). Our results were consistent with previous studies. We found Th1, Th2, and Th17 cells were more frequent in hemodialysis patients compared with healthy donors. And T cell proliferation and activation in response to antigen non-specific stimulation were weaker in hemodialysis patients. According to previous study, M-MDSCs exacted biological function in microenvironment instead of circulation (8). M-MDSCs in hemodialysis patients could not suppress T cells in circulation, indicated by its irrelevance with Th1, Th2, and Th17 cells and the risk of infectious diseases in our series studies (6). On the contrary, proinflammatory factors in the circulation of hemodialysis patients (32) induced M-MDSC accumulation and activation (24). Thus, hemodialysis related M-MDSCs shall not promote cardiovascular and cerebrovascular diseases by induction of immune suppression.

Hemodialysis-related M-MDSCs might promote atherosclerosis in the microenvironment of endothelial cells. We found that hemodialysis-related M-MDSCs displayed higher levels of cell surface adhesion molecules, which increased their capability to invade atherosclerotic lesions. M-MDSCs were reported to transform into macrophages in tumor microenvironment (9), which led to the speculation that M-MDSCs might be the major source of macrophages in atherosclerotic lesions. Additionally, hemodialysis related M-MDSCs were similar with the plasma of hemodialysis patients induced M-MDSC in the activity of arginase compared with their control, monocytes of health donor and plasma of health donors induced M-MDSC. Their exhaustion of local L-arginine by increased activity of arginase might be of great importance for the development of atherosclerosis lesions (21, 22). Moreover, we found migration of vascular endothelial cells, as a critical process for the development of atherosclerosis (23), was promoted by hemodialysis-related M-MDSCs through deprivation of L-arginine. Above all, hemodialysis related M-MDSC presented enhanced recruitment to atherosclerotic lesions, promoted the migration of endothelial cells through exhaustion of local L-arginine, which might be the mechanism of ESRD related atherosclerosis.

The latent causes for the induction of hemodialysis-related M-MDSCs were not clear. We found that M-MDSCs were elevated after hemodialysis (6). Plasma IFN-*γ*, TNF-α, and IL-6 were elevated in hemodialysis patients compared with healthy control. IL-6 was associated with the prognosis, which was similar with previous studies (33). Notably, linear regression found positive association between M-MDSC levels and IL-6 levels among hemodialysis patients. According to previous reports, IFN-*γ* and TNF-α induced the immune suppressive function of M-MDSC, with IL-6 causing M-MDSC expansion (24). The present study found that plasma of hemodialysis patients induced M-MDSCs significantly compared with plasma from health donors. Besides IL-6 neutralizing antibody partially abrogated the induction. Neutralizing antibody of IFN-*γ* and TNF-α partially decreased the generation of arginase of the induced M-MDSCs. Thus, our results indicated that the elevation of plasma IL-6 in the hemodialysis patients might be one of the causes of M-MDSC elevation, with the increase of IFN-*γ* and TNF-α associated with activation of M-MDSC.

Decreasing M-MDSC might contribute to less hemodialysis-related cardiovascular disease and stroke. Elimination of M-MDSC by specific antibodies was not practical due to lack of specific surface markers for human M-MDSCs. M-MDSC specific surface markers are needed to be identified in future study. A series of clinically available agents targeting MDSCs, such as all-transretinoic acid and cyclooxygenase 2 (COX2) inhibitors (8), might benefit patients with hemodialysis by suppressing MDSCs. However, the mechanisms of M-MDSC expansion and activation were diseases specific. It is necessary to investigate the mechanism of hemodialysis-related M-MDSCs. Besides, it is interesting to access the M-MDSC levels among hemodialysis patients taking all-transretinoic acid or COX2 inhibitors.

In summary, the present study found that M-MDSCs were elevated in ESRD patients under hemodialysis, and they exhibited a strong association with the risk of cardiovascular and cerebrovascular diseases. Hemodialysis related M-MDSC presented enhanced recruitment to atherosclerotic lesions, promoted the migration of endothelial cells through exhaustion of local L-arginine.

## Data Availability Statement

The original contributions presented in the study are included in the article/**Supplementary Material**; further inquiries can be directed to the corresponding authors.

## Ethics Statement

The studies involving human participants were reviewed and approved by the Clinical Ethics Review Board of the Third Affiliated Hospital of Guangzhou Medical University and the Third Affiliated Hospital of Sun Yat-sen University. The patients/participants provided their written informed consent to participate in this study.

## Author Contributions

XL and Y-FX designed and funded this study. Y-FX, J-RC, W-YZ, and C-ML conducted sample collection and quality control as well as in all experiments. Y-FX and C-ML followed up the patients. J-RC did statistical analysis. XL and Y-FX wrote this paper and all coauthors revised it. All authors contributed to the article and approved the submitted version.

## Funding

This study was supported by the National Natural Science Foundation of China (81972677 and 81700645), Natural Science Foundation of Guangdong (No. 2019A1515012198 and 2019A1515011187), Guangzhou Science and Technology Project (201904010461), the Fundamental Research Funds for the Central Universities (19ykpy17), and Tip-Top Scientific and Technology Innovative Youth Talents of Guangdong Special Support Program (2019TQ05Y266).

## Conflict of Interest

The authors declare that the research was conducted in the absence of any commercial or financial relationships that could be construed as a potential conflict of interest.
