# Supplementary material for: Expansion of Monocytic Myeloid-Derived Suppressor Cells in Patients Under Hemodialysis Might Lead to Cardiovascular and Cerebrovascular Events
Source: Front Immunol. 2021 Jan 29;11:577253. doi: 10.3389/fimmu.2020.577253 (PMC7878392; doi:10.3389/fimmu.2020.577253)
Supplement: Supplementary file 1 [file DataSheet_1.docx]

Supplementary data

Figure legend

**Figure S1**. Patient flow of the enrollment of end-stage renal disease (ESRD) patients under hemodialysis.

**Figure S2.** Venn diagram of the distribution of end-stage renal disease (ESRD) patients under hemodialysis with different monocytic myeloid derived suppressor cells (M-MDSCs) level.

**Figure S3.** Correlation of monocytic myeloid derived suppressor cells (M-MDSCs) with clinical characteristics in end-stage renal disease (ESRD) patients under hemodialysis.

**Figure S4.** Absolute cell number for T cell proliferation experiment. (A) Absolute cell numbers for CD8^+^ and CD4^+^ T cell count in monocyte from health donor and T cells coculture system. (B) Absolute cell numbers for CD8^+^ and CD4^+^ T cell count in monocytic myeloid-derived suppressor cells (M-MDSCs) from end-stage renal disease (ESRD) patients under hemodialysis and T cells coculture system. (C) Absolute cell numbers for CD8^+^ and CD4^+^ T cell count in monocytic myeloid-derived suppressor cells (M-MDSCs) from end-stage renal disease (ESRD) patients under hemodialysis and T cells coculture system under the administration of nor-NOHA, L-arginine, or L-NMMA.

**Figure S5** T helper (Th) cells in in end-stage renal disease (ESRD) patients under hemodialysis (HD) and health donor (Con). (A) Gating strategy of Th1, Th2 and Th17 cells by flow cytometry analysis. Th1 cells was defined as CD4^+^IFN-γ^+^. Th2 cells was defined as CD4^+^IL-4^+^ with Th17 cells defined as CD4^+^IL-17^+^. (B) Frequency of Th cells in CD4^+^ cells and their absolute counts in the peripheral blood of healthy controls (Con) and ESRD patients under hemodialysis (HD).

**Figure S6**. Effect of arginase inhibitor NOHA, L-arginine supplementation, or inducible nitric oxide synthase (iNOS) inhibitor L-NMMA on T cell proliferation. T cells from health donors were stimulated with anti-CD3 / anti-CD28 with treatments as indicated, evaluated for T cell proliferation by CFSE labeling, and IFN-γ production in supernatants by ELISA. Representative flow cytometry data, cumulative data, absolute cell number count, and concentration of IFN-γ in the media were shown (n = 3).

Table S1: antibodies used for flow cytometer analysis

| REAGENT or RESOURCE | SOURCE | IDENTFIER |
| --- | --- | --- |
| Antibodies |  |  |
| APC-conjugated anti-human HLA-DR antibody | eBioscience CA, USA | 17-9956-42 |
| Mouse IgG2b kappa Isotype Control (eBMG2b), APC | eBioscience CA, USA | Cat#17-4732-81 |
| FITC-conjugated anti-human CD11b antibody | eBioscience CA, USA | 11-0118-42 |
| Mouse IgG1 kappa Isotype Control (P3.6.2.8.1), FITC | eBioscience CA, USA | Cat#11-4714-81 |
| PE cy7-conjugated anti-human CD14 antibody | eBioscience, CA, USA | 25-0149-42 |
| Mouse IgG1 kappa Isotype Control (P3.6.2.8.1), PE-Cyanine7 | eBioscience CA, USA | Cat#25-4714-80 |
| eFlour450-conjugated anti-human CD15 antibody | eBioscience | Cat# 48-4752-80 |
| PE-conjugated anti-human CD4 antibody | TONBO | REF#50-0049-T025 |
| Mouse IgG2b, kappa | TONBO | REF# 50-4732-U100 |
| PE-conjugated anti-human CD3 antibody | Bioscience | Cat#12-0038-42 |
| APC-conjugated anti-human IL17 antibody | eBioscience CA, USA | Cat# 17-7179-42 |
| Mouse IgG1 kappa Isotype Control (P3.6.2.8.1), APC | eBioscience CA, USA | Cat#17-4714-82 |
| FITC-conjugated anti-human IFN-γ antibody | eBioscience CA, USA | Cat# 17-7319-82 |
| [Mouse IgG1 kappa Isotype Control (P3.6.2.8.1), FITC](https://www.thermofisher.com/antibody/product/11-4714-81) | eBioscience CA, USA | Cat#11-4714-81 |
| PE-Cy7-conjugated anti-human IL-4 antibody | eBioscience CA, USA | Cat# 25-7049-82 |
| Mouse IgG1 kappa Isotype Control (P3.6.2.8.1), PE-Cyanine7 | eBioscience CA, USA | Cat# 25-4714-80 |
| APC-conjugated anti-human CD8 antibody | TONBO | REF#20-0088-T100 |

| Table S2: antibodies used for ELISA | | |  |
| --- | --- | --- | --- |
| REAGENT or RESOURCE | SOURCE | IDENTFIER | |
| Antibodies |  |  | |
| IFN-γ | Dakewei Bioengineering Co., Shenzhen, Guangdong | DKW12-1000-09 | |
| hTNF-α | CST, MA, USA | #8902SC | |
| IL-6 | CST, MA, USA | #8904SC | |

| Table S3 Primers for qRT-PCR | | |
| --- | --- | --- |
|  | Forward Primer | Reverse Primer |
| CXCR4 | GGGCAATGGATTGGTCATCCT | TGCAGCCTGTACTTGTCCG |
| VLA-4 | TACAGATGCAGGATCGGAAAGA | AGGTTCTCCATTAGGGCTACC |
| PSGL-1 | TGTTGCTGATCCTACTGGGC | CACAGTGGTAGACTCAGGGGT |
| L-selectin | ACCCAGAGGGACTTATGGAAC | GCAGAATCTTCTAGCCCTTTGC |
| P-selectin | CTGTTACCCTGGATTCTATGGGC | GCTGCACTGCGAGTTAAAAGA |
| E-selectin | AGAGTGGAGCCTGGTCTTACA | CCTTTGCTGACAATAAGCACTGG |
| GAPDH | ACAACTTTGGTATCGTGGAAGG | GCCATCACGCCACAGTTTC |

| Table S4. The characteristics of ESRD patients under hemodialysis with different M-MDSC levels | | | |  |
| --- | --- | --- | --- | --- |
| Characteristics | M-MDSC low  N=41 | M-MDSC Int  N=21 | M-MDSC high  N=42 | *P** |
| Age (year, mean, SD) | 56.0±15.5 | 61.1±15.3 | 59.8±13.3 | 0.475 |
| Male (n, %) | 21 (51.2%) | 9 (42.9%) | 24 (57.1%) | 0.381 |
| Primary disease (n, %) |  |  |  | 0.516 |
| Hypertension | 4 (9.8%) | 2 (9.5%) | 3 (7.1%) |  |
| Diabetes mellitus | 6 (14.6%) | 3 (33.3%) | 17 (40.5%) |  |
| Glomerulonephritis | 5 (12.2%) | 0 (0%) | 2 (4.8%) |  |
| Systemic lupus erythematosus | 4 (9.8%) | 3 (14.3%) | 4 (9.5%) |  |
| Obstructive nephropathy | 0 (0%) | 2 (9.5%) | 2 (4.8%) |  |
| Unknown | 21 (51.2%) | 6 (28.6%) | 13 (31.0%) |  |
| Others | 1 (2.4%) | 1 (4.8%) | 1 (2.4%) |  |
| Acute dialysis (n, %) | 34 (82.9%) | 15 (71.4%) | 33 (78.6%) | 0.955 |
| Characteristics before dialysis |  |  |  |  |
| Hemoglobin (g/L, mean, SD) | 72.1±16.5 | 79.4±22.2 | 76.1±17.4 | 0.682 |
| BUN (mmol/L, median, range) | 34.7 (12.8-68.9) | 27.7 (13.5-63.5) | 27.8 (12.2-55.8) | 0.191 |
| Creatinine (μmol/L, median, range) | 904 (305-2214) | 853 (175-2062) | 780 (185-1875) | 0.082 |
| Albumin (g/L, mean, SD) | 34.3±5.3 | 33.2±5.4 | 32.5±6.5 | 0.234 |
| Calcium serum (mmol/L, median, range) | 2.08 (1.28-2.36) | 2.03 (1.17-2.26) | 2.03 (1.04-2.81) | 0.778 |
| Serum phosphorus (mmol/L, mean, SD) | 1.85 (1.13-3.56) | 2.00 (1.24-3.36) | 1.89 (1.21-3.41) | 0.691 |
| eGFR (ml/min per 1.73m^2^, median, range) | 1.62 (0.42-7.75) | 1.90 (0.45-12.19) | 4.18 (0.53-10.86) | 0.041 |
| LDL-C (mmol/L, media, range) | 2.6 (1.0-6.1) | 2.9 (1.7-5.0) | 3.1 (1.1-5.9) | 0.434 |
| HDL-C (mmol/L, median, range) | 1.1 (0.5-2.6) | 1.1 (0.6-2.2) | 1.0 (0.4-2.6) | 0.371 |
| Triglyceride (mmol/L, median, range) | 1.4 (0.40-3.8) | 1.4 (0.5-5.8) | 1.5 (0.6-3.4) | 0.298 |
| Cholesterol (mmol/L, median, range) | 4.3 (2.9-7.5) | 4.7 (3.1-6.4) | 4.9 (2.5-9.1) | 0.686 |
| Characteristics at M-MDSC testing |  |  |  |  |
| Dialysis history | 15.2 (1.0-93.3) | 10.1 (1.0-76.1) | 7.6 (0.9-107.5) | 0.176 |
| Hemoglobin (g/L, median, range) | 105 (63-149) | 107 (56-127) | 102 (70-165) | 0.518 |
| BUN (mmol/L, mean, SD) | 27.8±6.8 | 25.4±5.8 | 23.7±7.6 | 0.022 |
| Creatinine (μmol/L, median, range) | 1055 (503-1779) | 1069 (481-2095) | 885 (310-1728) | 0.001 |
| Albumin (g/L, mean, SD) | 37.6±2.8 | 37.7±2.6 | 36.8±5.4 | 0.370 |
| Calcium serum (mmol/L, mean, SD) | 2.27±0.29 | 2.13±0.17 | 2.20±0.22 | 0.613 |
| Serum phosphorus (mmol/L, median, range) | 2.13 (0.58-3.84) | 2.04(1.19-2.74) | 1.86 (0.85-3.67) | 0.215 |
| Parathormone (pg/mL, median, range) | 324.8 (9.4-3000) | 265.8 (29.8-1631.4) | 245.0 (36.8-3000) | 0.101 |
| Urea reduction rate (median, range) | 69.0 (50-95.3) | 68.1 (53.5-87.7) | 67.2 (44.5-88.5) | 0.215 |
| White blood cell (10^9^/L, mean, SD) | 6.42±1.93 | 6.24±0.81 | 6.97±1.76 | 0.074 |
| Neutrophils (10^9^/L, mean, SD) | 4.47±1.65 | 4.32±0.86 | 4.71±1.44 | 0.317 |
| Lymphocyte (10^9^/L, mean, SD) | 1.29±0.53 | 1.10±0.37 | 1.42±0.48 | 0.042 |
| Monocyte (10^9^/L, mean, SD) | 0.37±0.14 | 0.40±0.10 | 0.50±0.18 | 0.001 |
| Red blood cell (10^9^/L, mean, SD) | 3.53±0.59 | 3.59±0.80 | 3.48±0.68 | 0.573 |
| LDL-C (mmol/L, mean, SD) | 2.51±0.99 | 2.55±0.82 | 2.33±0.91 | 0.302 |
| HDL-C (mmol/L, mean, SD) | 1.14±0.47 | 1.23±0.64 | 1.11±0.60 | 0.611 |
| Triglyceride (mmol/L, median, range) | 1.40 (0.54-8.98) | 1.29 (0.36-2.52) | 1.25 (0.53-9.22) | 0.926 |
| Cholesterol (mmol/L, median, range) | 4.04 (1.37-8.49) | 4.32(2.67-6.17) | 4.07 (2.23-6.52) | 0.569 |
| Complications |  |  |  |  |
| Diabetes mellitus | 11 (26.8%) | 6 (28.6%) | 19 (45.2%) | 0.061 |
| Coronary heart disease | 9 (22.0%) | 5 (23.8%) | 17 (40.5%) | 0.050 |
| Chronic heart failure | 23 (56.1%) | 16 (76.2%) | 33 (78.6%) | 0.089 |
| Cerebrovascular disease | 14 (34.1%) | 8 (38.1%) | 18 (42.9) | 0.448 |
| Death cases | 11 (26.8) | 6 (28.6) | 20 (47.6) | 0.035 |
| Cause |  |  |  | 0.204 |
| Heart disease | 2 (18.2) | 1 (16.7) | 4 (20.0) |  |
| Cerebrovascular disease | 5 (45.5) | 1 (16.7) | 12 (60.0) |  |
| Infectious disease | 1(9.1) | 2 (33.3) | 3 (15.0) |  |
| Others | 3 (27.3) | 2 (33.3) | 1 (5.0) |  |

Abbreviation: M-MDSC, monocytic myeloid suppressor cell; Int, intermediate; ESRD, end stage renal disease; SD, standard deviation; BUN, Blood urine nitrogen; LDL-C, low density lipoprotein-cholesterol; HDL-C, high density lipoprotein-cholesterol; * Parameters display by mean and SD were compared by t test and those displayed by median and range were compared by nonparametric Mann-Whitney U tests; Comparison of patients in High group with others.

| Table S5. The characteristics of health control testing MDSC level | |
| --- | --- |
| Characteristics | N=60 |
| Age (year, mean, SD) | 57.6±15.7 |
| Male (n, %) | 30 (50.0%) |
| Hypertension | 20 (33.3%) |
| Diabetes mellitus | 13 (21.7%) |
| Coronary heart disease | 13 (21.7%) |
| Chronic heart failure | 0 (0.0%) |
| Cerebrovascular disease | 14 (23.3%) |

Abbreviation: MDSC, myeloid suppressor cell

Table S6. Cox analysis of prognostic factors for overall survival of hemodialysis patients

|  | *P* | HR | 95%CI | |
| --- | --- | --- | --- | --- |
| Gender | .711 | 1.209 | .444 | 3.290 |
| Age | .009 | 1.050 | 1.012 | 1.090 |
| Hemodialysis history | .086 | .979 | .955 | 1.003 |
| White blood cell | .697 | .735 | .156 | 3.463 |
| Neutrophils | .695 | 1.358 | .294 | 6.266 |
| Lymphocyte | .627 | 1.620 | .231 | 11.345 |
| Monocyte | .180 | 14.225 | .294 | 687.927 |
| Hemoglobin | .820 | .995 | .953 | 1.039 |
| Red blood cell | .968 | 1.024 | .315 | 3.333 |
| BUN | .277 | .957 | .884 | 1.036 |
| Creatinine | .306 | 1.001 | .999 | 1.003 |
| Albumin | .514 | .957 | .838 | 1.093 |
| Calcium serum | .724 | .695 | .092 | 5.267 |
| Serum phosphorus | .770 | .865 | .329 | 2.279 |
| Parathormone | .325 | 1.000 | 1.000 | 1.001 |
| LDL-C | .726 | .886 | .452 | 1.740 |
| HDL-C | .695 | .809 | .280 | 2.334 |
| TRIG | .979 | .994 | .634 | 1.559 |
| CHOL | .308 | 1.460 | .705 | 3.024 |
| Urea reduction rate | .235 | .970 | .923 | 1.020 |
| IFN-γ | .852 | 1.000 | .997 | 1.002 |
| TNF-α | .228 | 1.003 | .998 | 1.008 |
| IL-6 | .782 | .976 | .824 | 1.157 |
| PMN-MDSC | .761 | 1.004 | .978 | 1.031 |
| M-MDSC | .040 | 1.011 | 1.001 | 1.023 |

Abbreviation: PMN-MDSC, polymorphonuclear- myeloid suppressor cell; M-MDSC, monocytic myeloid suppressor cell; HR, hazard ratio; CI, confidence interval; BUN, Blood urine nitrogen; LDL-C, low density lipoprotein-cholesterol; HDL-C, high density lipoprotein-cholesterol; IFN, interferon; TNF, tumor necrosis factor.

Table S7 Cox analysis of prognostic factors for acute myocardial infarction events of hemodialysis patients

|  | *P* | HR | 95%CI | |
| --- | --- | --- | --- | --- |
| Gender | .097 | 1.240E+31 | .000 | 6.808E+067 |
| Age | .045 | 8.940 | 1.053 | 75.872 |
| Hemodialysis history | .058 | 6.223 | .941 | 41.166 |
| White blood cell | .754 | .000 | .000 | 9109 E+26 |
| Neutrophils | .998 | .869 | .000 | 5.007E+038 |
| Lymphocyte | .212 | 4.561E+31 | .000 | 2.102E+081 |
| Monocyte | .966 | .019 | .000 | 3.882E+076 |
| Hemoglobin | .350 | 7.045 | .118 | 421.466 |
| Red blood cell | .325 | .000 | .000 | 7.411E+33 |
| BUN | .089 | 663.584 | .373 | 1.181E+6 |
| Creatinine | .576 | .923 | .697 | 1.223 |
| Albumin | .697 | 78.024 | .000 | 2.711E+11 |
| Calcium serum | .880 | .000 | .000 | 1.495E+090 |
| Serum phosphorus | .194 | .000 | .000 | 5.787E+31 |
| Parathormone | .268 | .985 | .958 | 1.012 |
| LDL-C | .861 | .002 | .000 | 5.970 E+28 |
| HDL-C | .392 | 1.159 E+23 | .000 | 6.601E+075 |
| TRIG | .955 | .313 | .000 | 1.536 E+17 |
| CHOL | .404 | 2.083 E+10 | .000 | 3.513E+34 |
| Urea reduction rate | .390 | 14.658 | .032 | 6646.096 |
| PMN-MDSC | .340 | .514 | .131 | 2.018 |
| M-MDSC | .785 | .891 | .390 | 2.037 |
| IFN-γ | .027 | 1.331 | 1.033 | 1.715 |
| TNF-α | .065 | 1.408 | .979 | 2.025 |
| IL-6 | .541 | 8.145 | .010 | 6800.604 |

Abbreviation: PMN-MDSC, polymorphonuclear- myeloid suppressor cell; M-MDSC, monocytic myeloid suppressor cell; HR, hazard ratio; CI, confidence interval; BUN, Blood urine nitrogen; LDL-C, low density lipoprotein-cholesterol; HDL-C, high density lipoprotein-cholesterol; IFN, interferon; TNF, tumor necrosis factor.

Table S8 Cox analysis of prognostic factors for heart failure survival of hemodialysis patients

|  | *P* | HR | 95%CI | |
| --- | --- | --- | --- | --- |
| Gender | .686 | .817 | .306 | 2.181 |
| Age | .148 | 1.033 | .989 | 1.079 |
| Hemodialysis history | .062 | 1.024 | .999 | 1.050 |
| White blood cell | .137 | 4.215 | .632 | 28.133 |
| Neutrophils | .075 | .169 | .024 | 1.200 |
| Lymphocyte | .166 | .200 | .020 | 1.953 |
| Monocyte | .072 | .017 | .000 | 1.441 |
| Hemoglobin | .366 | .978 | .932 | 1.026 |
| Red blood cell | .473 | 1.556 | .466 | 5.200 |
| BUN | .549 | 1.032 | .931 | 1.144 |
| Creatinine | .032 | .997 | .994 | 1.000 |
| Albumin | .039 | 1.166 | 1.008 | 1.348 |
| Calcium serum | .371 | 2.548 | .328 | 19.775 |
| Serum phosphorus | .654 | .778 | .259 | 2.332 |
| Parathormone | .112 | .999 | .998 | 1.000 |
| LDL-C | .059 | 2.678 | .964 | 7.443 |
| HDL-C | .110 | 2.393 | .821 | 6.968 |
| TRIG | .208 | 1.372 | .839 | 2.243 |
| CHOL | .100 | .399 | .133 | 1.193 |
| Urea reduction rate | .046 | 1.058 | 1.001 | 1.119 |
| PMN-MDSC | .656 | 1.008 | .974 | 1.043 |
| M-MDSC | .288 | 1.007 | .994 | 1.020 |
| IFN-γ | .445 | .999 | .996 | 1.002 |
| TNF-α | .163 | .996 | .990 | 1.002 |
| IL-6 | .118 | 1.177 | .960 | 1.444 |

Abbreviation: PMN-MDSC, polymorphonuclear- myeloid suppressor cell; M-MDSC, monocytic myeloid suppressor cell; HR, hazard ratio; CI, confidence interval; BUN, Blood urine nitrogen; LDL-C, low density lipoprotein-cholesterol; HDL-C, high density lipoprotein-cholesterol; IFN, interferon; TNF, tumor necrosis factor.

Table 9 Cox analysis of prognostic factors for stroke events of hemodialysis patients

|  | *P* | HR | 95%CI | |
| --- | --- | --- | --- | --- |
| Gender | .387 | 1.768 | .486 | 6.437 |
| Age | .010 | 1.058 | 1.014 | 1.105 |
| Hemodialysis history | .156 | 1.020 | .992 | 1.049 |
| White blood cell | .029 | 10.820 | 1.281 | 91.408 |
| Neutrophils | .032 | .093 | .011 | .820 |
| Lymphocyte | .138 | .148 | .012 | 1.850 |
| Monocyte | .002 | .000 | .000 | .040 |
| Hemoglobin | .393 | .971 | .909 | 1.038 |
| Red blood cell | .788 | 1.228 | .275 | 5.491 |
| BUN | .550 | 1.040 | .914 | 1.184 |
| Creatinine | .336 | .998 | .995 | 1.002 |
| Albumin | .308 | .934 | .820 | 1.064 |
| Calcium serum | .921 | .894 | .097 | 8.245 |
| Serum phosphorus | .464 | 1.565 | .472 | 5.189 |
| Parathormone | .364 | 1.000 | .999 | 1.001 |
| LDL-C | .881 | .921 | .314 | 2.698 |
| HDL-C | .169 | .409 | .114 | 1.461 |
| TRIG | .364 | .720 | .354 | 1.465 |
| CHOL | .518 | 1.437 | .479 | 4.313 |
| Urea reduction rate | .118 | 1.049 | .988 | 1.114 |
| PMN-MDSC | .744 | .995 | .963 | 1.027 |
| M-MDSC | .000 | 1.042 | 1.025 | 1.060 |
| IFN-γ | .048 | .997 | .993 | 1.000 |
| TNF-α | .074 | .995 | .989 | 1.001 |
| IL-6 | .086 | .821 | .655 | 1.028 |

Abbreviation: PMN-MDSC, polymorphonuclear- myeloid suppressor cell; M-MDSC, monocytic myeloid suppressor cell; HR, hazard ratio; CI, confidence interval; BUN, Blood urine nitrogen; LDL-C, low density lipoprotein-cholesterol; HDL-C, high density lipoprotein-cholesterol; IFN, interferon; TNF, tumor necrosis factor.
