# Supplementary figures and images for: Expansion of Monocytic Myeloid-Derived Suppressor Cells in Patients Under Hemodialysis Might Lead to Cardiovascular and Cerebrovascular Events
Source: Front Immunol. 2021 Jan 29;11:577253. doi: 10.3389/fimmu.2020.577253 (PMC7878392; doi:10.3389/fimmu.2020.577253)

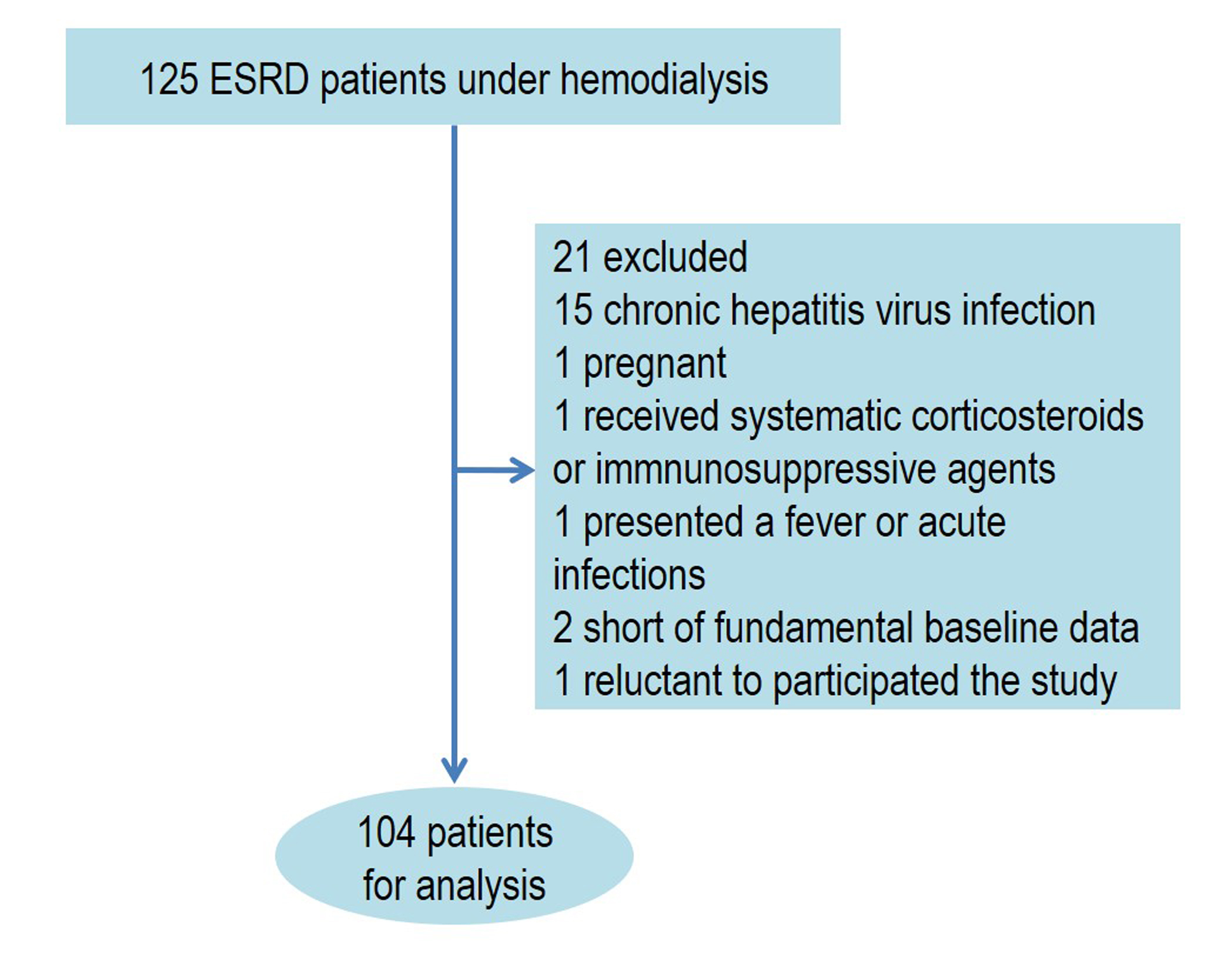

Supplement: Supplementary file 2 [file Image_1.jpeg]

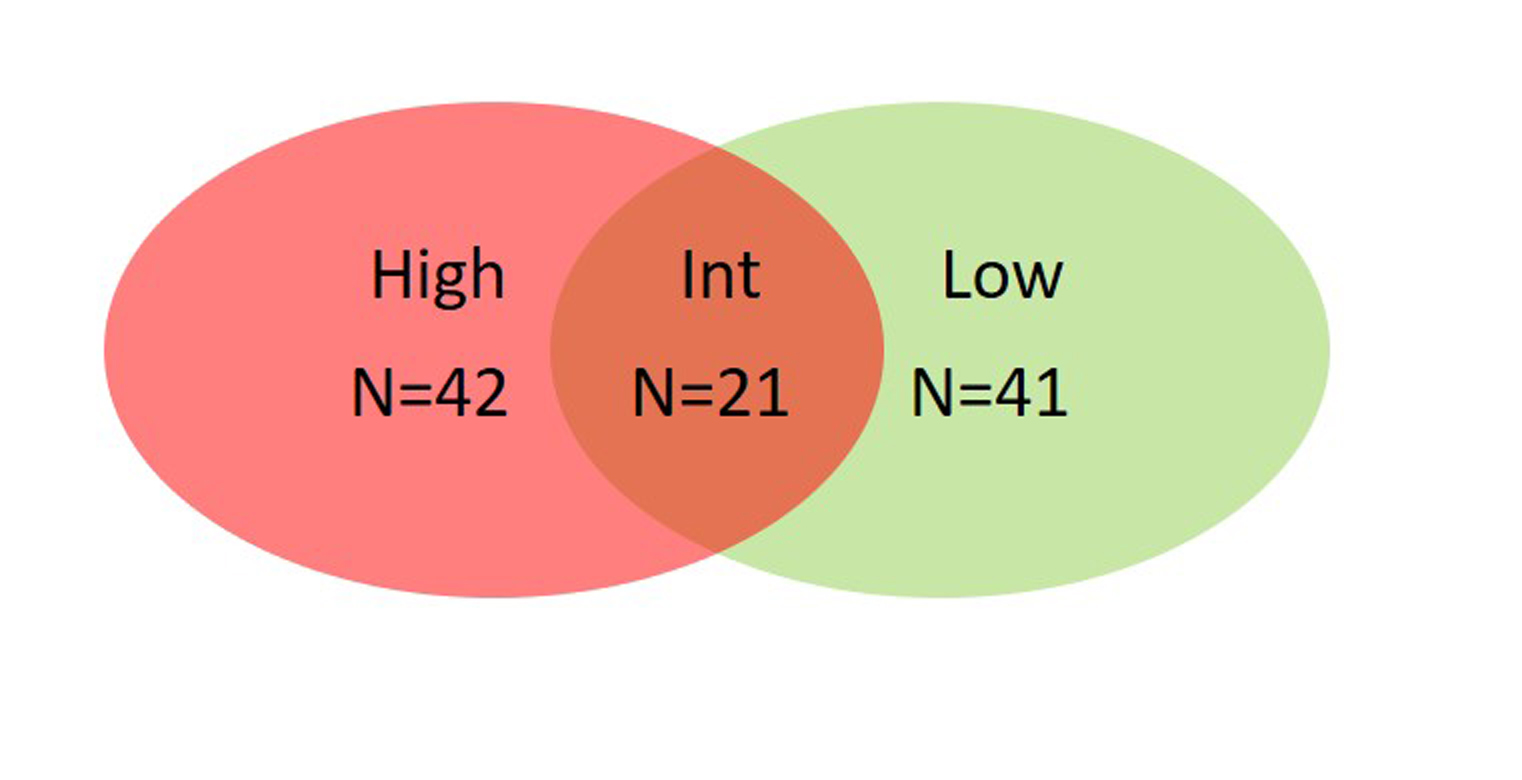

Supplement: Supplementary file 3 [file Image_2.jpeg]

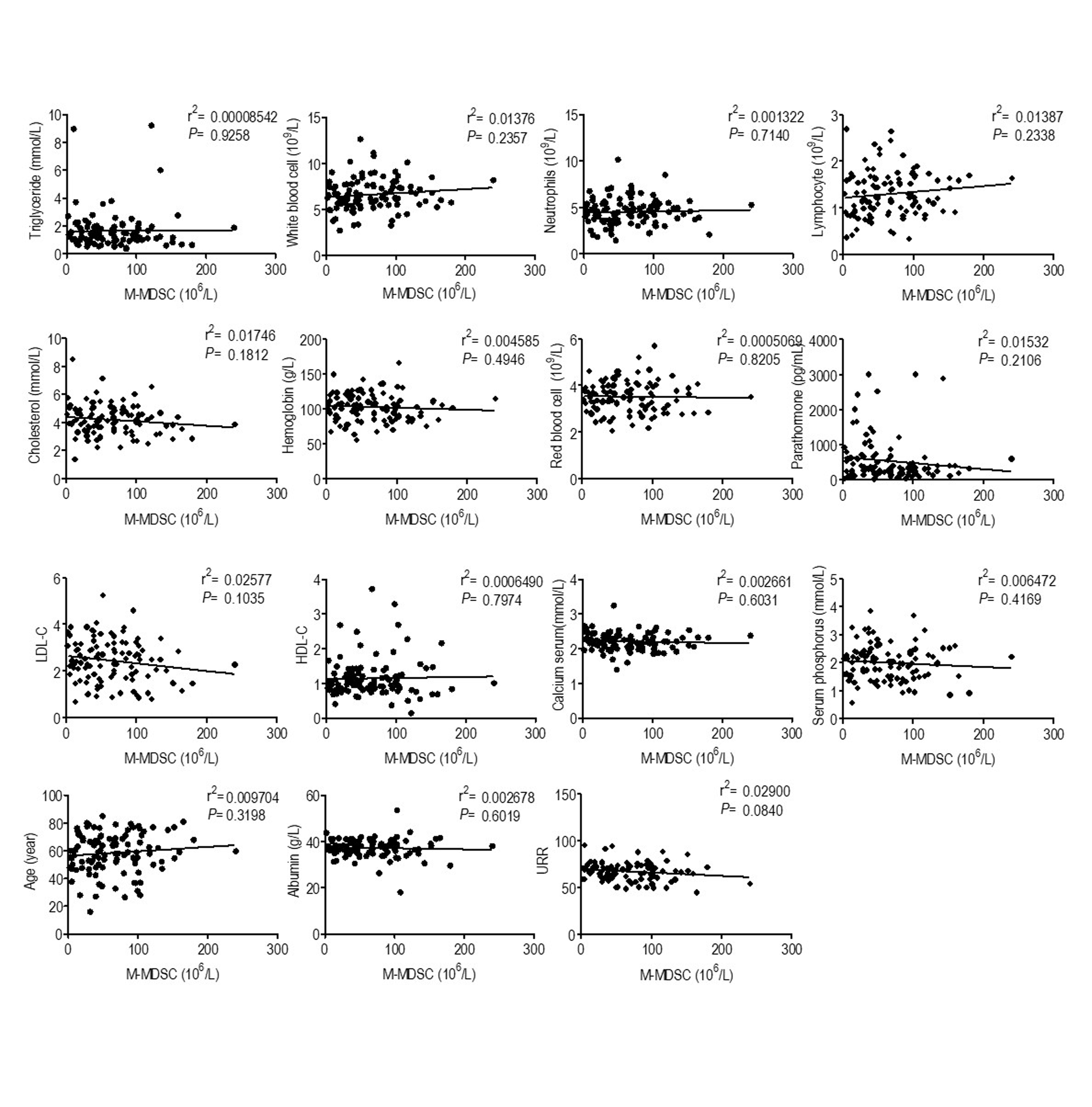

Supplement: Supplementary file 4 [file Image_3.jpeg]

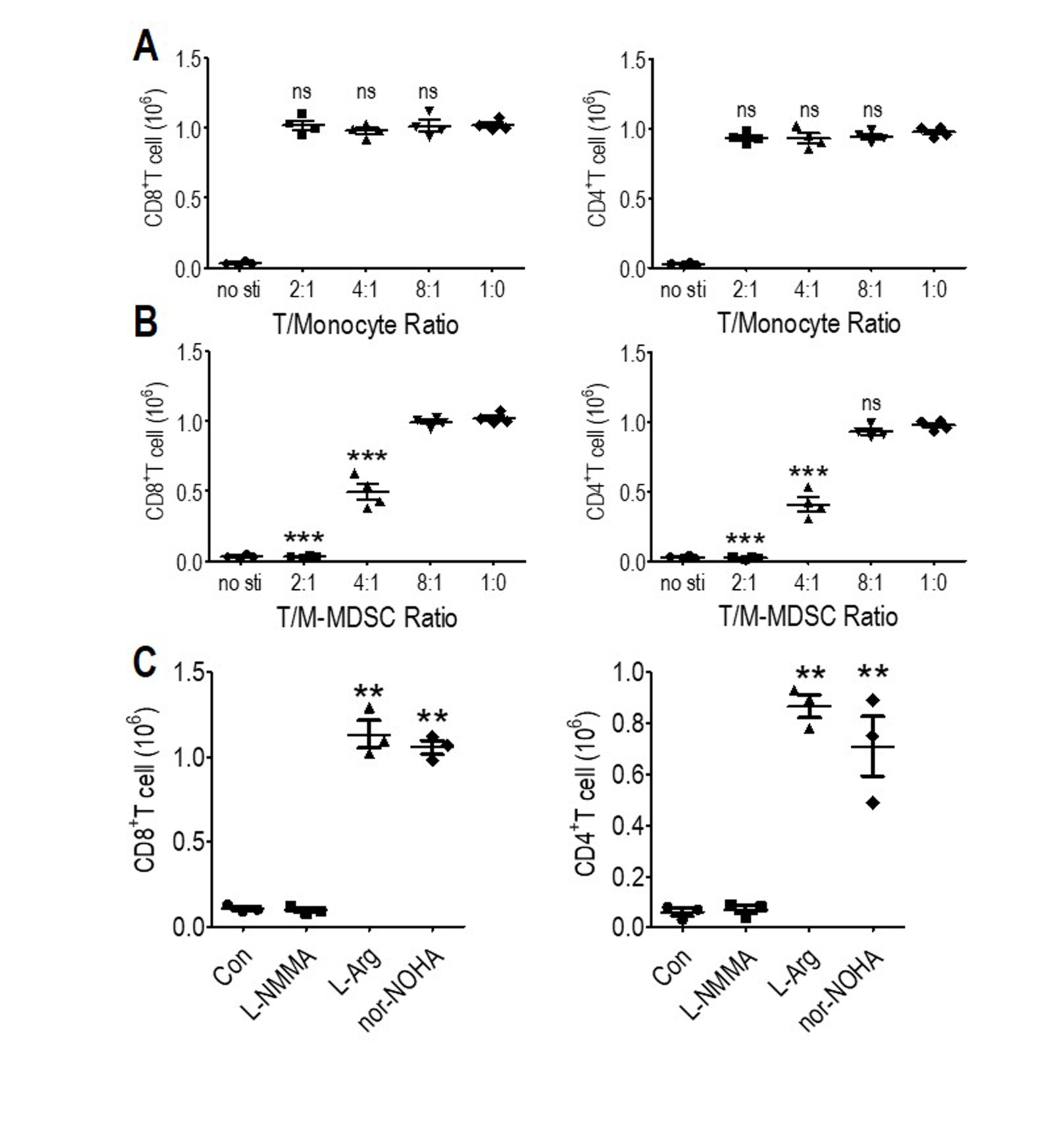

Supplement: Supplementary file 5 [file Image_4.jpeg]

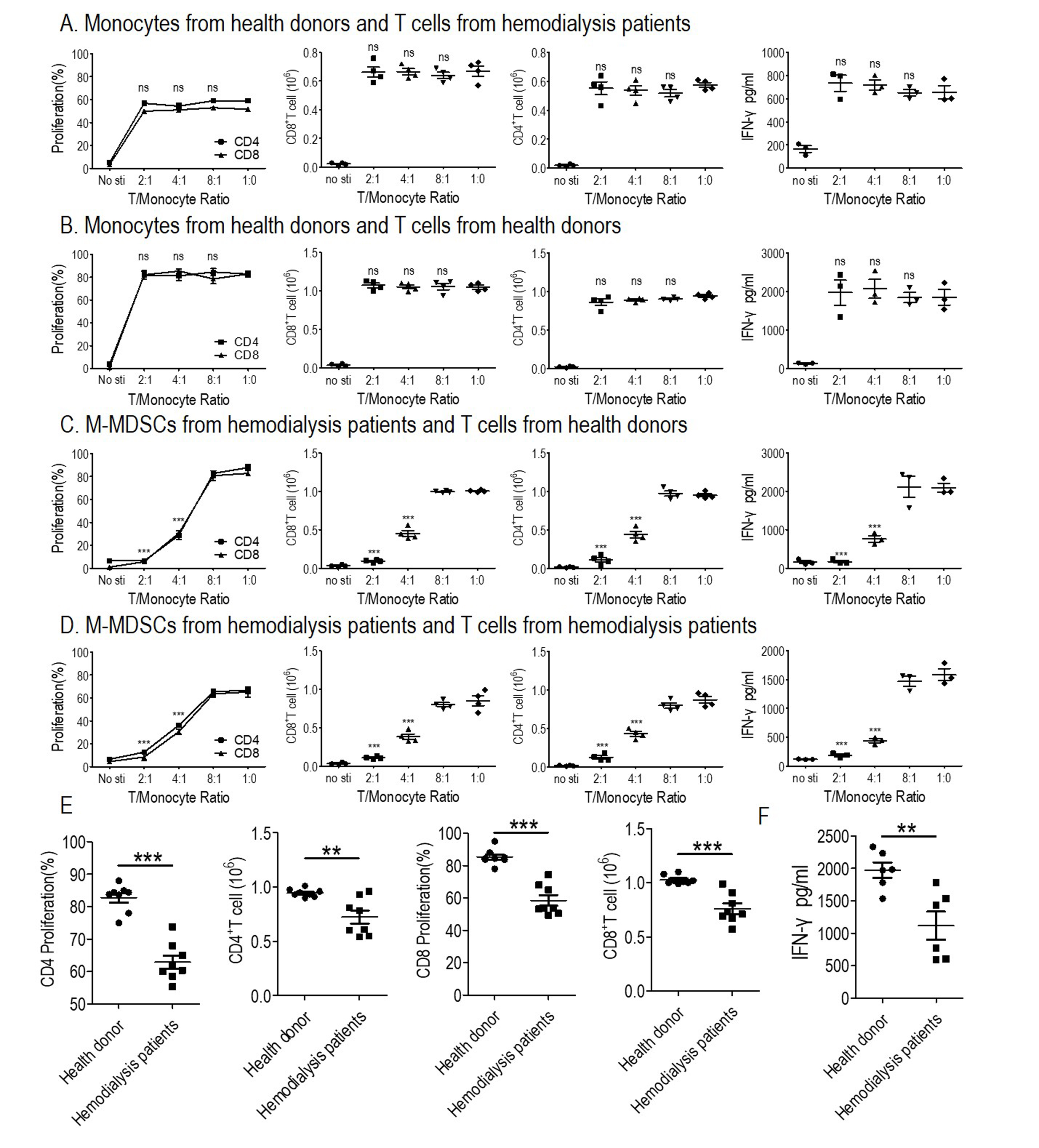

Supplement: Supplementary file 6 [file Image_5.jpeg]

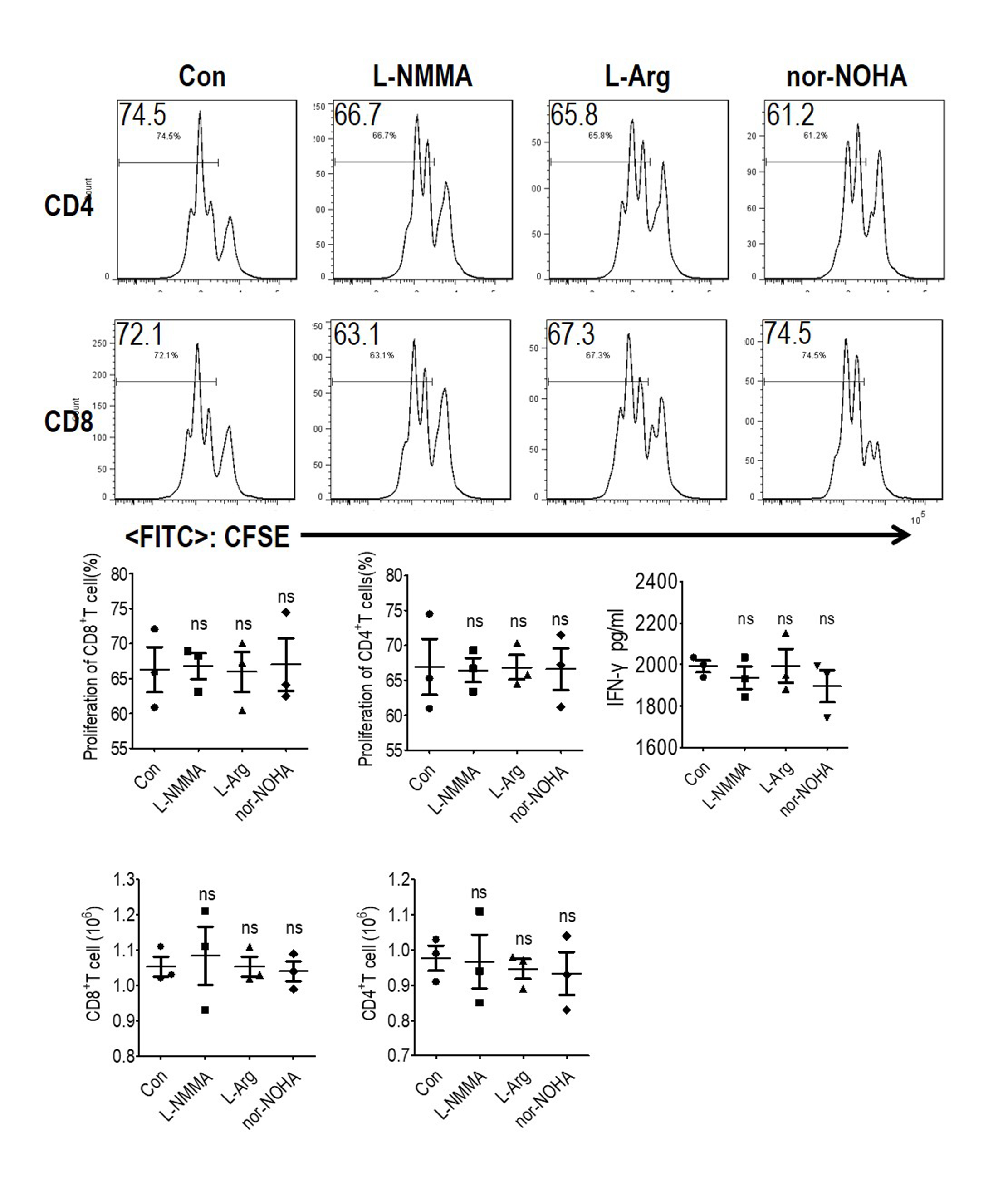

Supplement: Supplementary file 7 [file Image_6.jpeg]

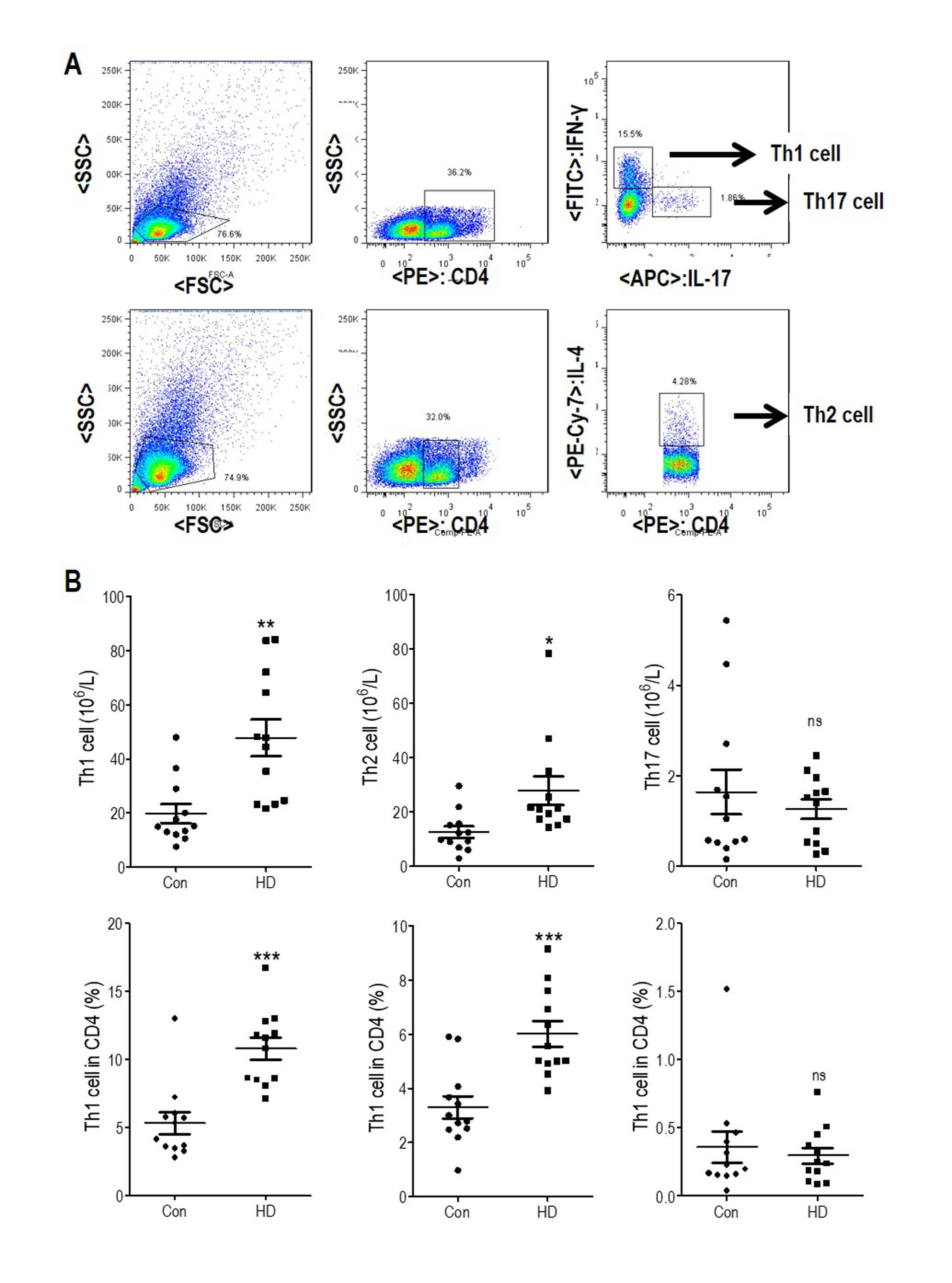

Supplement: Supplementary file 8 [file Image_7.jpeg]
